# Remdesivir Inhibits Tubulointerstitial Fibrosis in Obstructed Kidneys

**DOI:** 10.3389/fphar.2021.626510

**Published:** 2021-07-02

**Authors:** Lin Xu, Bo Tan, Di Huang, Meijie Yuan, Tingting Li, Ming Wu, Chaoyang Ye

**Affiliations:** ^1^ Department of Nephrology, Shuguang Hospital Affiliated to Shanghai University of Traditional Chinese Medicine, Shanghai, China; ^2^ TCM Institute of Kidney Disease of Shanghai University of Traditional Chinese Medicine, Shanghai, China; ^3^ Key Laboratory of Liver and Kidney Diseases, Ministry of Education, Shanghai, China; ^4^ Shanghai Key Laboratory of Traditional Chinese Clinical Medicine, Shanghai, China; ^5^ Clinical Pharmacokinetic Laboratory, Shuguang Hospital Affiliated to Shanghai University of Traditional Chinese Medicine, Shanghai, China; ^6^ Department of Nephrology, The First Hospital of Hebei Medical University, Shijiazhuang, China

**Keywords:** remdesivir (GS-5734), renal fibrosis, COVID-19, CKD-chronic kidney disease, obstructed kidneys

## Abstract

**Aim:** Kidney impairment is observed in patients with COVID-19. The effect of anti-COVID-19 agent remdesivir on kidneys is currently unknown. We aimed to determine the effect of remdesivir on renal fibrosis and its downstream mechanisms.

**Methods:** Remdesivir and its active nucleoside metabolite GS-441524 were used to treat TGF-β stimulated renal fibroblasts (NRK-49F) and human renal epithelial (HK2) cells. Vehicle or remdesivir were given by intraperitoneal injection or renal injection through the left ureter in unilateral ureteral obstruction (UUO) mice. Serum and kidneys were harvested. The concentrations of remdesivir and GS-441524 were measured using LC-MS/MS. Renal and liver function were assessed. Renal fibrosis was evaluated by Masson’s trichrome staining and Western blotting.

**Results:** Remdesivir and GS-441524 inhibited the expression of fibrotic markers (fibronectin and aSMA) in NRK-49F and HK2 cells. Intraperitoneal injection or renal injection of remdesivir attenuated renal fibrosis in UUO kidneys. Renal and liver function were unchanged in remdesivir treated UUO mice. Two remdesivir metabolites were detected after injection. Phosphorylation of Smad3 that was enhanced in cell and animal models for renal fibrosis was attenuated by remdesivir. In addition, the expression of Smad7, an anti-fibrotic factor, was increased after remdesivir treatment *in vitro* and *in vivo*. Moreover, knockdown of Smad7 blocked the antifibrotic effect of GS and RDV on renal cells.

**Conclusion:** Remdesivir inhibits renal fibrosis in obstructed kidneys.

## Introduction

A novel coronavirus (2019-nCoV) reported in Wuhan in late December 2019 has rapidly spread to the rest of the world (Zhou et al., 2020). Coronavirus disease 2019 (COVID-19) is becoming a worldwide public health event due to the rapid increase in new cases and the high severity and mortality (Wang C. et al., 2020; Wang D. et al., 2020).

Chronic kidney disease (CKD) is a common disorder and the prevalence of CKD is around 10% in adults (Elliott et al., 2017; Jager et al., 2017). Patients with CKD might be more vulnerable to COVID-19 because a recent study shows that COVID-19 patients in the intensive care unit were more likely to have comorbidities (72.2 vs. 37.3%) than patients not in the intensive care unit (Wang D. et al., 2020). On the other hand, 2019-nCoV also attack kidneys beside inducing pneumonia (Wang T. et al., 2020). It has been found that angiotensin-converting enzyme 2 (ACE2), which mediates the entry of 2019-nCoV into human cells is highly expressed in renal tubular cells, implying that 2019-nCoV may directly bind to ACE2-positive cells in the kidney and thus induce kidney injuries (Fan et al., 2020). Indeed, a clinical study reported that 27.06% of patients with COVID-19 exhibited acute renal failure (ARF), and elderly patients (≥60 years) were more likely to develop ARF (65.22 vs. 24.19%) (Diao et al., 2020). A further immunohistochemistry analysis revealed that the antigen for 2019-nCoV accumulates in renal tubules (Diao et al., 2020). Another clinical study with 59 COVID-19 patients showed that proteinuria occurred in 63% of patients (Li et al., 2020). 19 and 27% COVID-19 patients have elevated plasma creatinine and urea nitrogen levels, respectively, (Li et al., 2020). A consecutive cohort study with 710 COVID-19 patients further shows that the prevalence of renal impairment is high, which is associated with in-hospital death (Cheng et al., 2020).

Renal interstitial fibrosis is a common pathway and main pathological basis for the progression of various chronic kidney diseases to the end-stage renal disease (ESRD) (Gewin et al., 2017; Hewitson et al., 2017). It is characterized by excessive deposition of extracellular matrix in the kidney leading to completely loss of renal function (Gewin et al., 2017; Hewitson et al., 2017). Loss of renal tubule drives the development of renal interstitial fibrosis by producing a large number of profibrotic factors such as TGF-β (Gewin et al., 2017; Liu et al., 2018). It has been shown by several animal models that the TGF-β/Smad signaling pathway plays a key role in renal fibrosis (Zhou et al., 2010; Zhang et al., 2015). Smad3 is pro-fibrotic, however Smad2 and Smad7 are anti-fibrotic in the kidney. (Chen et al., 2018).

Remdesivir (GS-5734, RDV) is a nucleoside analogue designed for the treatment of severe acute respiratory syndrome coronavirus (SARS), the Middle East respiratory syndrome (MERS) and Ebola virus (Warren et al., 2016; Sheahan et al., 2020). It can be rapidly anabolized to the active triphosphate metabolite and then incorporated into the newly synthesized RNA strand of the virus as a substrate for viral RNA-dependent RNA synthetase (RdRp), thereby prematurely terminating viral RNA transcription (Warren et al., 2016; Sheahan et al., 2020). *In vitro* study shows that RDV can effectively inhibit the infection of 2019-nCoV (Wang M. et al., 2020). A single case study shows that clinical condition of the first severely infected COVID-19 patient in the United States was improved after administration of RDV in 24 h (Holshue et al., 2020). In a further large double-blind, randomized, placebo-controlled trial, RDV shortened the recovery time of COVID-19 patients (Beigel et al., 2020). Currently, RDV is the only drug approved by USFDA to treat COVID-19 patients (Saha et al., 2020). However, considering the potential toxicity, RDV is contraindicated in patients with low glomerular filtration rate. (Adamsick et al., 2020).

Whether RDV treatment is beneficial to kidneys, especially to already injured kidneys is currently unknown. We hypothesized that RDV inhibits renal interstitial fibrosis through Smad3.

## Materials and Methods

### Animals and UUO Operation

Male C57 mice (C57bl/6j background, SPF grade, 20–25 g) were purchased and housed in Shanghai Model Organisms Center Inc. (SMOC) according to local regulations and guidelines.

After anesthesia with sodium pentobarbital (8 mg/kg, i.p.), the left mouse kidney was exposed by an incision. UUO operation was performed through twice ligation of the left ureter with 4–0 nylon sutures. Animal experiments described here in were approved by the animal experimentation ethics committee of Shanghai University of Traditional Chinese Medicine (PZSHUTCM18111601).

For the experiment by intraperitoneal (i.p.) injection, mice were randomly divided into four groups: 1) Sham + vehicle (*n* = 5), 2) Sham + RDV (*n* = 7), 3) UUO + vehicle (*n* = 8), and 4) UUO + RDV (*n* = 8) group.

In a previous study (Sheahan et al., 2017), a dose of 25 mg/kg RDV (subcutaneously injection) was chosen for pharmacokinetic analysis in mice, and the anti-virus effect of RDV was observed in mice at this dosage. Since the bioavailability through i.p. injection is normally higher than subcutaneously injection, thus 10 mg/kg of RDV was used for i.p. injection in this study. Mice were treated with vehicle or RDV daily and were sacrificed at day 10 at 1 h after last injection. Serum and kidney tissues were collected.

For the experiment by intrarenal injection, mice were randomly divided into two groups: 1) UUO + vehicle (*n* = 11) and 2) UUO + RDV (*n* = 11) group. Four mice from each group were sacrificed at 1 h after renal injection, and the rest of mice were sacrificed at day 7. Serum and kidney tissues were collected. Alanine transferase (ALT), aspartate aminotransferase (AST), blood urea nitrogen (BUN) and serum creatinine (Scr) values were assessed in clinical laboratory of Shuguang hospital using routine methods.

### Intrarenal Drug Administration

RDV (Product name GS-5734; Cat. No. CSN19703) was purchased from CSNpharm (Chicago, Illinois, United States) and dissolved in DMSO as a 50 mg/ml stock, which was further diluted into normal saline by sonication as a working solution. 0.04% typan blue dye (A601140, Sangon, Shanghai, China) was added into vehicle or RDV working solution to monitor the injection process. 50 μl of vehicle or RDV (1 mg/ml) was injected retrogradely once into the left kidney via the ureter using a sterile 26-gauge needle (0.45 mm × 16 mm). Right after the injection, unilateral ureteral obstruction was performed.

### Cell Culture

HK2 renal proximal tubular epithelial cells were obtained from the Cell Bank of Shanghai Institute of Biological Sciences (Chinese Academy of Science). NRK-49F rat kidney interstitial fibroblast cells were purchased from National Infrastructure of Cell Line Resource, Chinese Academy of Medical Sciences. HK2 and NRK-49F cells were cultured in DMEM/F12 medium containing 10%FBS and 0.5% penicillin/streptomycin in an atmosphere of 5% CO2 and 95% air at 37°C. For Western blotting, HK2 and NRK-49F cells were seeded in 6-well plate to 40–50% confluence, which were starved overnight with DMEM/F12 medium containing 0.5% fetal bovine serum. In the next day, fresh medium containing 0.5% fetal bovine serum was changed, and then cells were exposed to 2.5 ng/ml TGF-β (Peprotech, Rocky Hill, NJ, United States) for 24 h or 48 h in the presence of various concentration of GS-441524 (Catalog No. T7222, Targetmol, Boston, MA, United States) or RDV (Product name GS-5734; Cat. No. CSN19703; CSNpharm, Chicago, IN, United States). The concentration of GS-441524 (GS) and RDV used in the *in vitro* study were chosen based on previous studies (Warren et al., 2016; Murphy et al., 2018; Sheahan et al., 2020; Wang M. et al., 2020).

### siRNA Transfection

NRK-49F cells plated in six-well culture dishes and were cultured in DMEM/F12 medium containing 10% FBS and 0.5% penicillin/streptomycin in an atmosphere of 5% CO2 and 95% air at 37°C. When cells reached 50–60% confluence, siRNA was transfected using ExFect 2,000 (Vazyme, Nanjing, China) according to the manufacturer’s instruction. Cells were cultured in DMEM/F12 medium containing 10% FBS for 24 h and protein was extracted. For interventional study, cells were refreshed with 0.5% FBS medium in the second day and treated with GS or RDV in the presence of 2.5 ng/ml TGF-β (Peprotech, Rocky Hill, NJ, United States) for another 24h for protein extraction.

The siRNA sequences were as follows: nonsense control (NC) forward, 5’-UUCUCC GAACGUGUCACGUTT-3’ and reverse, 5’-ACG​UGA​CAC​GUU​CGG​AGA​ATT-3’; rat Smad7 forward, 5’-UGG​CAU​ACU​GGG​AGG​AGA​ATT-3’ and reverse, 5’-UUCU CCU​CCC​AGU​AUG​CCA​TT-3’.

### Quantitation of RDV and Its Two Metabolites

RDV and its two metabolites, alanine metabolite (Ala-Met) and nucleoside metabolite (GS), in serum and kidney were determined using a LC-MS/MS method as described in a previous literature with minor revision (Warren et al., 2016). In brief, 200 ul of serum or kidney homogenates were mixed with equivalent volume of acetonitrile-methanol mixture (1: 1, v/v). Then, internal standards were added, vortexed, and centrifuged at 15,000 g for 5 min. The supernatant was collected and mixed with equivalent volume of deionized water. An aliquot of 10 μl was subsequently injected into a Waters LC-MS/MS system which contains an ACQUITY UPLC and a Xevo TQ-S tandem quadrupole mass spectrometry (Waters, Milford, MA, United States). Standard solutions of RDV and GS were used to plot calibration curves for quantification. Due to the standard is commercially unavailable, alanine metabolite was semi-determined by mass spectrometry response.

### Masson’s Trichrome Staining and Quantification

Mouse kidneys were fixed in 4% paraformaldehyde and further embedded in paraffin. Masson’s trichrome staining was performed using a standard protocol. Briefly, the Four-μm-thick sections of paraffin-embedded kidney tissue was stained with hematoxylin, and then with ponceau red liquid dye acid complex, which was followed by incubation with phosphomolybdic acid solution. Finally, the tissue was stained with aniline blue liquid and acetic acid. Images were obtained with the use of a microscope (Nikon 80i, Tokyo, Japan).

The collagen positive area was quantified using the ImageJ software. The color threshold (the Hue was set to “125–220”; the saturation was “0–255” and the brightness was “150–225”) was set up to measure the area of collagen fibers stained with blue dye. The total area was measured under the threshold mode “0–205”. Five fields of view were captured from each pathological section clockwise. The blue positive area was divided by the total area for each field, and the average value was calculated for each mouse section.

### Western Blotting Analysis

Renal protein was extracted from the cortex of mouse kidneys. The protein concentration was measured by the Bradford method, and the supernatant was dissolved in 5x SDS-PAGE loading buffer (P0015L, Beyotime Biotech, Nantong, China). Samples were subjected to SDS-PAGE gels. After electrophoresis, proteins were electro-transferred to a polyvinylidene difluoride membrane (Merck Millipore, Darmstadt, Germany), which was incubated in the blocking buffer (5% non-fat milk, 20 mM Tris-HCl, 150 mM NaCl, PH = 8.0, 0.01%Tween 20) for 1 h at room temperature and was followed by incubation with anti-fibronectin (1:1,000, ab23750, Abcam), anti-pSmad3 (1:1,000, ET1609-41, HUABIO), Smad2/3 (1:500, sc-133098, Santa Cruz), Smad7 (1:1,000, AB37036, aboci), Smad7 (1:1,000, P102346, KleanAB), anti-Collagen I (1:500, sc-293182, Santa Cruz), anti-α-SMA (1:1,000, ET1607-53, HUABIO), anti-GAPDH (1:5,000, 60,004-1-lg, Proteintech), or anti-α-tubulin (1:1,000, AF0001, Byotime) antibodies overnight at 4°C. Binding of the primary antibody was detected by an enhanced chemiluminescence method (BeyoECL Star, P0018A, Byotime) using horseradish peroxidase-conjugated secondary antibodies (goat anti-rabbit IgG, 1:1,000, A0208, Beyotime or goat anti-mouse IgG, 1:1,000, A0216, Beyotime). The quantification of protein expression was performed using -Image J.

### Statistical Analysis

Results were presented as mean ± SD.Differences among multiple groups were analyzed by one-way analysis of variance (ANOVA) and two-way ANOVA according to the experimental settings and comparison between two groups was performed by unpaired student t-test by using GraphPad Prism version 8.0.0 for Windows (GraphPad Software, San Diego, California United States). A *p* value of lower than 0.05 was considered statistically significant.

## Results

### RDV Inhibited Renal Fibrosis *in Vitro*

**FIGURE 1:**
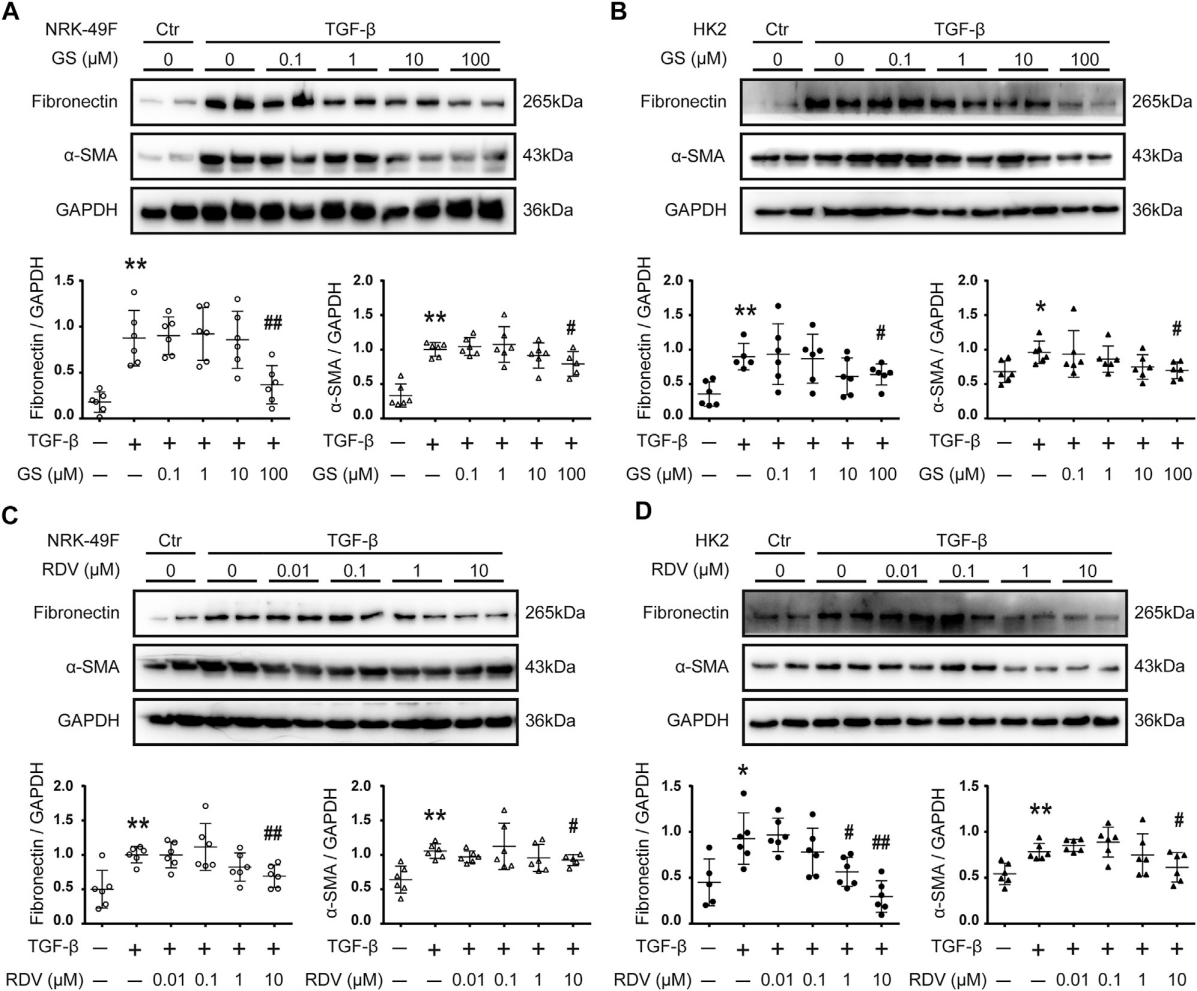
Remdesivir and its intermediate metabolite GS-441524 inhibited renal fibrosis *in vitro* **(A)** Following TGF-β stimulation, NRK-49F rat renal fibroblasts cells were treated with various concentration (0, 0.1, 1, 10, 100 μM) of GS-441524 (GS) for 24 h. The expression of fibronectin (FN) and alpha-smooth muscle actin (*α*-SMA) in NRK-49F cells were analyzed by Western blotting and quantified **(B)** Upon TGF-β stimulation, HK2 human renal epithelial cells were treated with various concentration (0, 0.1, 1, 10, 100 μM) of GS for 48 h. The expression of FN and α-SMA in HK2 cells were analyzed by Western blotting and quantified **(C)** TGF-β stimulated NRK-49F cells were treated with various concentration (0, 0.01, 0.1, 1, 10 μM) of Remdesivir (RDV) for 24 h. The expression of FN and α-SMA in NRK-49F cells were analyzed by Western blotting and quantified **(D)** TGF-β stimulated HK2 cells were treated with various concentration (0, 0.01, 0.1, 1, 10 μM) of RDV for 48 h. The expression of FN and *α*-SMA in HK2 cells were analyzed by Western blotting and quantified. Data represent mean ± SD. *p < 0.05 vs. Vehicle-DMSO; **p < 0.01 vs. Vehicle-DMSO; #*p* < 0.05 vs. TGF-β-DMSO; ##*p* < 0.01 vs. TGF-β-DMSO. One representative result of at least three independent experiments is shown.

GS, the active metabolite of RDV, was used to treat TGF-β stimulated rat renal interstitial fibroblasts (NRK-49F) and human renal epithelial (HK2) cells (Agostini et al., 2018). The protein expression of fibronectin (FN) and alpha smooth muscle actin (α-SMA) were assessed by Western blotting as markers for fibrosis. Stimulation with 2.5 ng/ml TGF-β for 24 h increased the expression of FN and α-SMA in NRK-49F cells, and inhibition of these fibrotic markers by GS was observed at 100 μM (Figure 1A). The effect of GS on renal fibrosis was further studied using HK2 cells. HK2 cells were stimulated with 2.5 ng/ml TGF-β for 48 h, and the expression of FN and α-SMA were increased which were inhibited by GS at 100 μM (Figure 1B).

We further tested the direct effect of RDV on fibrosis *in vitro*. Down-regulation of FN and α-SMA expression by 24 h treatment of RDV was observed at 10 μM in TGF-β stimulated NRK-49F cells (Figure 1C). Figure 1D shows that 48 h treatment with RDV significantly inhibited the expression of FN and α-SMA at 1 μM or 10 μM, respectively, in TGF-β stimulated HK2 cells.

### Intraperitoneal Injection of RDV Inhibited Renal Fibrosis in UUO Mice

**FIGURE 2:**
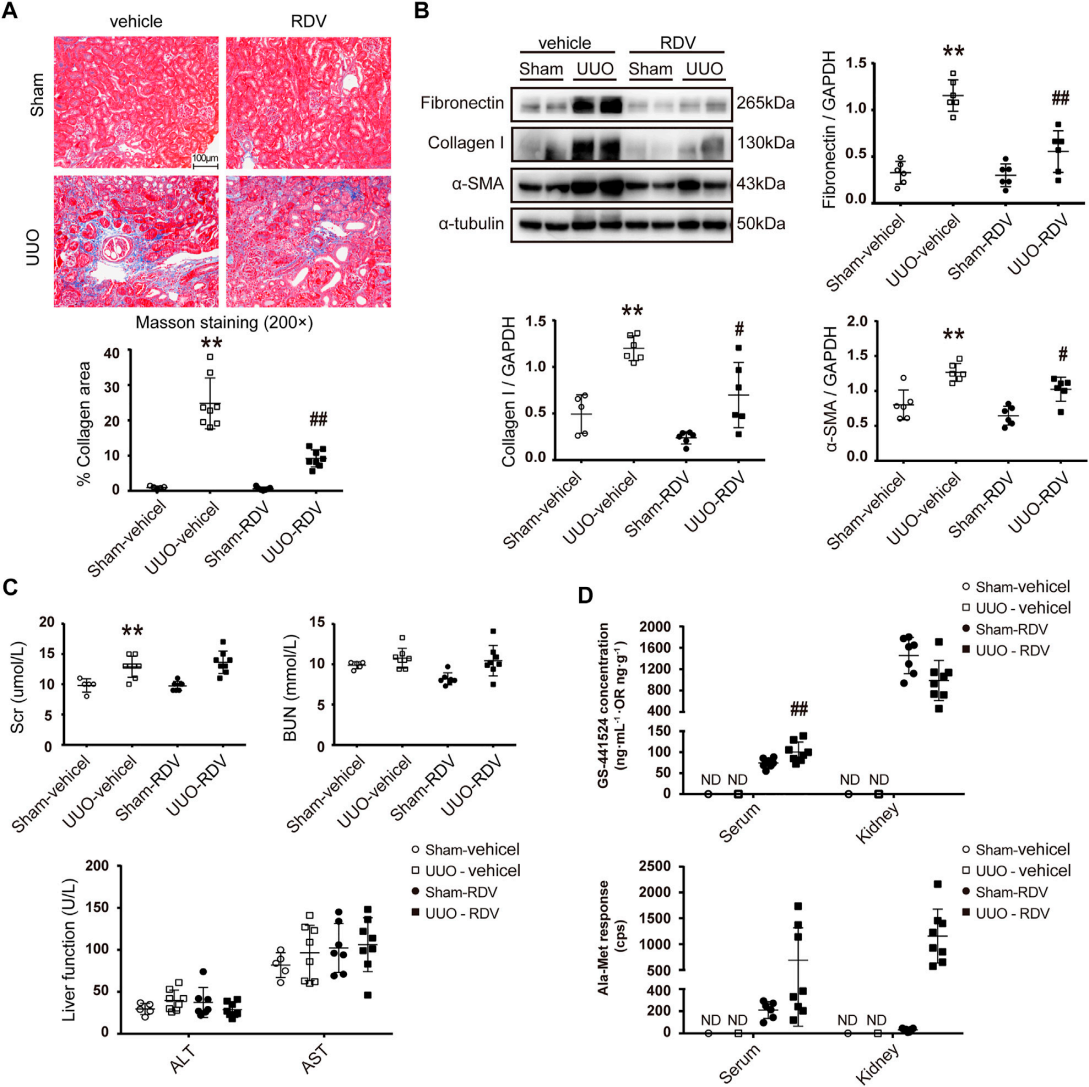
Intraperitoneal (i.p.) administration of RDV inhibited fibrosis in UUO mice.After sham or UUO operation, wide type c57 mice were treated with 10 mg/kg/d RDV by i.p. injection for 10 days. Serum and kidney tissues were collected 1 h after RDV injection at day 10 **(A)** Renal fibrosis was assessed by Masson’s trichrome staining, and then was quantified **(B)** The expression of FN, collagen I (Col-1) and α-SMA were analyzed by Western blotting. One representative of at least three independent experiments is shown **(C)** Renal function (Scr and BUN) and liver function (ALT and AST) were assessed **(D)** The concentrations of nucleoside metabolite (GS) and alanine metabolite (Ala-Met), two RDV metabolites, in serum and kidneys were determined by LC-MS/MS. Data represent mean ± SD. ND represents not determined. **p < 0.01 vs. Sham-vehicle; #*p* < 0.05 vs. UUO-vehicle; ##*p* < 0.01 vs. UUO-vehicle.

Mouse renal fibrosis model was induced by UUO operation. One day after sham or UUO operations, mice were treated with vehicle or RDV for 10 days. Treatment with RDV had no effect on body weight of sham and UUO mice, and all mice were survived during the treatment (data not shown). Mild interstitial fibrosis was observed in vehicle treated UUO mice, which was significantly attenuated by RDV (Figure 2A). The protein expression of FN, collagen-I (Col-I) and α-SMA were up-regulated in UUO mouse kidneys as compared with that in sham operated mouse kidneys, and the treatment with RDV significantly reduced the expression of these pro-fibrotic proteins in UUO mouse kidneys (Figure 2B). Liver function (ALT and AST) and renal function (Scr and BUN) were determined. RDV has no effect on either liver function or renal function (Figure 2C). Serum and kidney concentration of RDV and two metabolites of RDV (GS and Ala-Met) were determined by LC-MS/MS. RDV can not be detected in serum or kidney in both sham and UUO mice (data not shown). However, GS can be detected in the serum and kidney of RDV treated sham or UUO mice (Figure 2D). Similarly, serum or kidney Ala-Met can be detected in RDV treated sham or UUO mice (Figure 2D).

### Renal Injection of RDV Inhibited Renal Fibrosis in UUO Mice

**FIGURE 3:**
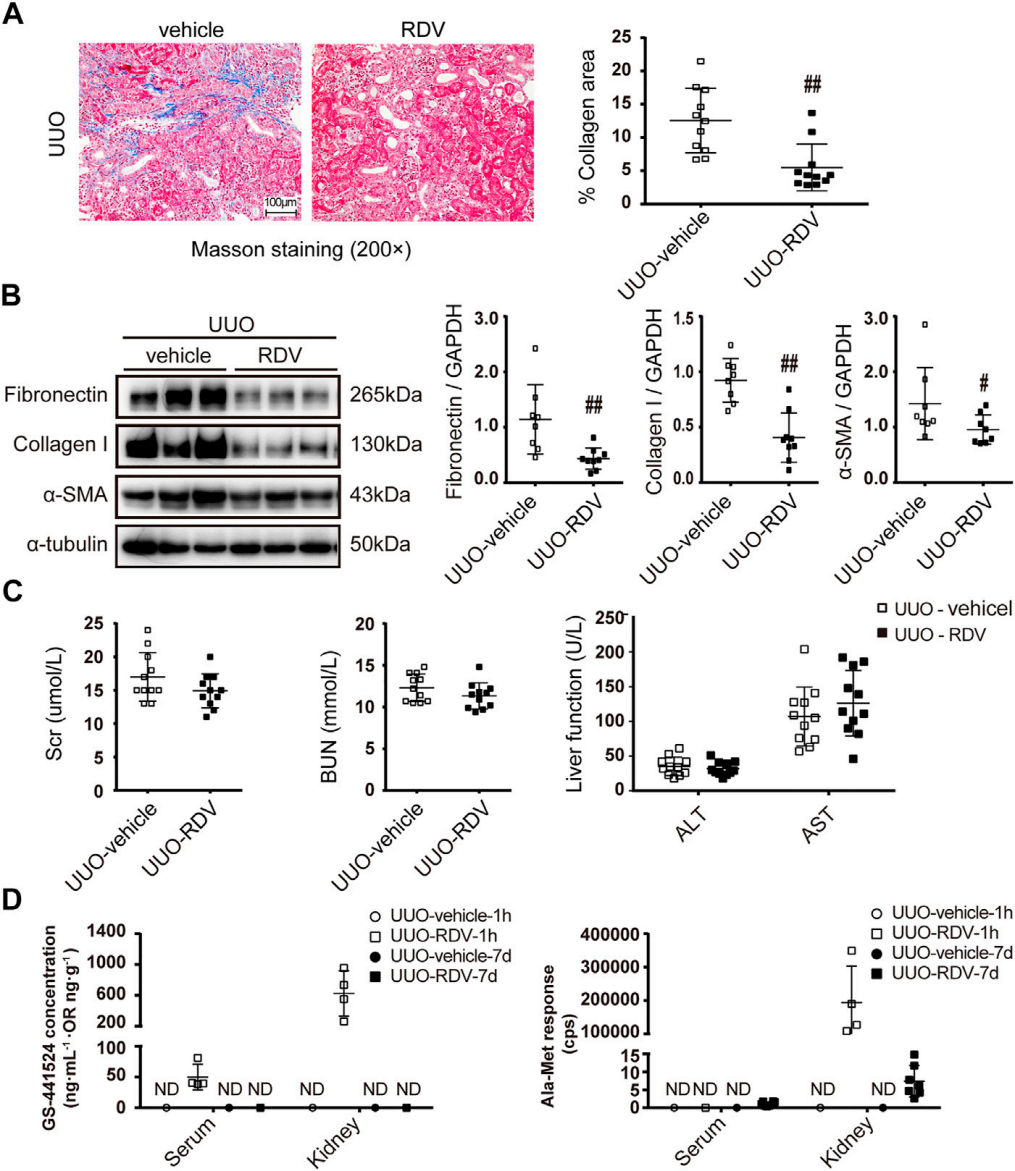
Intrarenal administration of RDV inhibited fibrosis in UUO mice. 50μL of vehicle or RDV (1 mg/ml) was injected intrarenally to the left kidney, which was subjected to the UUO operation thereafter. Serum and renal tissues were collected at 1 h or day 7 after UUO operation **(A)** Renal fibrosis was assessed by Masson’s trichrome staining, and then was quantified **(B)** The expression of FN, collagen I (Col-1) and *α*-SMA were measured by Western blotting. One representative of at least three independent experiments is shown **(C)** Liver function (ALT and AST) and renal function (Scr and BUN) were assessed **(D)** The concentrations of nucleoside metabolite (GS) and alanine metabolite (Ala-Met), two RDV metabolites, in serum and kidneys were determined by LC-MS/MS. Data represent mean ± SD. ND represents not determined. #*p* < 0.05 vs. UUO-vehicle; ##*p* < 0.01 vs. UUO-vehicle.

RDV (1 mg/ml, 50 μl/mouse) or vehicle was infused retrogradely through ureter to the left kidney which was subjected to unilateral utero ligation (UUO) operation. Masson staining shows that interstitial fibrosis was significantly attenuated in UUO kidneys at day 7 by local RDV injection (Figure 3A). The expression of FN, Col-I and α-SMA were reduced in RDV treated UUO kidneys as compared with that in vehicle treated UUO kidneys at day 7 as shown by Western blotting in Figure 3B. Liver function (ALT and AST) and renal function (Scr and BUN) were not changed by RDV in UUO mice (Figure 3C). The RDV metabolite GS can be detected in serum and kidney at 1 h after injection only in RDV treated mice, which can not be detected at day 7 (Figure 3D). The Ala-Met is abundant in RDV treated kidneys at 1 h after injection and it was reduced to background level at day 7 (Figure 3D).

### Phosphorylation of Smad3 was Attenuated by RDV in Cell and Animal Models for Renal Fibrosis

**FIGURE 4:**
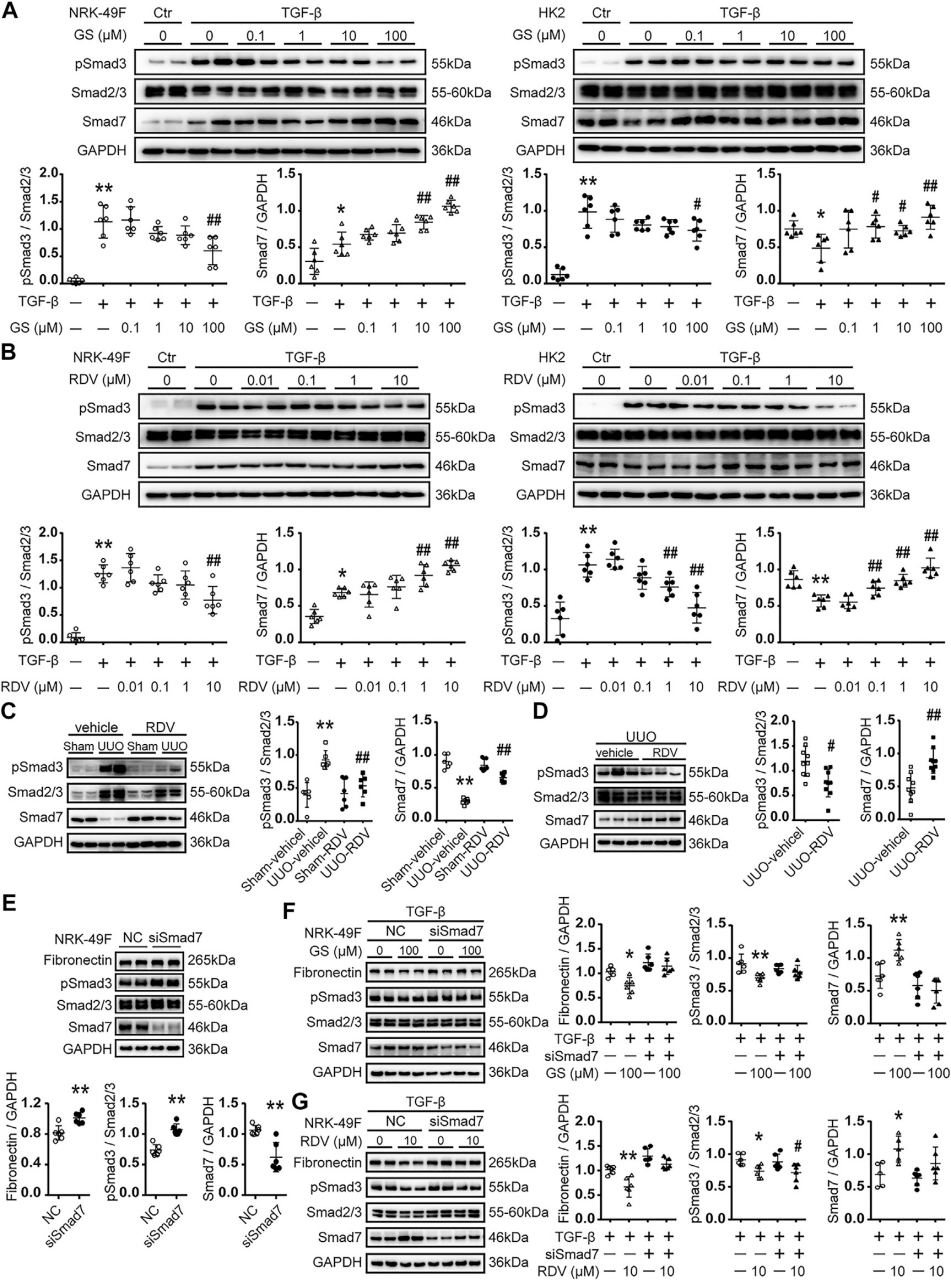
The expression of Smad7 and phosphorylation of Smad3 was regulated by RDV *in vitro* and *in vivo* **(A)** Following TGF-β stimulation, NRK-49F cells or HK2 were treated with various concentration of GS for 24 or 48 h, respectively. The expression of Smad7 and phosphorylated Smad3 (pSmad3) were analyzed by Western blotting and quantified **(B)** TGF-β stimulated NRK-49F or HK2 cells were treated with various concentration of RDV for 24 or 48 h, respectively. The expression of Smad7 and pSmad3 were analyzed by Western blotting and quantified. Data represent mean ± SD. **p < 0.01 vs. Vehicle-DMSO; #*p* < 0.05 vs. TGF-β-DMSO; ##*p* < 0.01 vs. TGF-β-DMSO. One representative result of at least three independent experiments is shown **(C)** After sham or UUO operation, wide type c57 mice were treated with 10 mg/kg/d RDV by i.p. injection for 10 days. The expression of Smad7 and pSmad3 was measured by Western blotting. One representative of at least three independent experiments is shown **(D)** 50 μl of vehicle or RDV (1 mg/ml) was injected intrarenally to the left kidney, which was subjected to the UUO operation thereafter. The expression of Smad7 and pSmad3 was measured by Western blotting at day 7. Data represent mean ± SD. ND represents not determined. **p < 0.01 vs. Sham-vehicle; #*p* < 0.05 vs. UUO-vehicle; ##*p* < 0.01 vs. UUO-vehicle **(E)** NRK-49F cells were transfected with nonsense control (NC) or rat Smad7 siRNA in 10% FBS medium. Cell lysates were collected at 24 h after stimulation. The expression of FN, Smad7 and pSmad3 were analyzed by Western blotting and quantified. Data represent mean ± SD. **p < 0.01 vs. NC **(F)** NRK-49F cells were transfected with nonsense control (NC) or rat Smad7 siRNA in 10% FBS medium. After 24 h, cells were refreshed with 0.5% FBS medium and treated with 100 μM GS in the presence of 2.5 ng/ml TGF-β for another 24 h. The expression of FN, Smad7 and pSmad3 were analyzed by Western blotting and quantified **(G)** NRK-49F cells were transfected with nonsense control (NC) or rat Smad7 siRNA in 10% FBS medium. After 24 h, cells were refreshed with 0.5% FBS medium and treated with 10 μM RDV in the presence of 2.5 ng/ml TGF-β for another 24 h. The expression of FN, Smad7 and pSmad3 were analyzed by Western blotting and quantified. Data represent mean ± SD. *p < 0.05 vs. TGF-β-NC; **p < 0.01 vs. TGF-β-NC; #*p* < 0.05 vs. TGF-β-siSmad7. One representative of at least three independent experiments is shown.

Stimulation with 2.5 ng/ml TGF-β for 24 h or 48 h increased the phosphorylation of Smad3 in NRK-49F or HK2 cells (Figure 4A). GS significantly attenuated the phosphorylation of Smad3 at 100 μM in NRK-49F cells or in HK2 cells (Figure 4A). RDV significantly reduced the expression of phosphorylated Smad3 (pSmad3) at 10 μM in NRK-49F or at 1 μM in HK2 cells (Figure 4B).

Phosphorylation of Smad3 was increased at day 10 after UUO operation in mouse kidneys, which was reduced by intraperitoneal injection of RDV (Figure 4C). Similarly, renal injection of RDV inhibited the expression of pSmad3 in UUO mouse kidneys (Figure 4D).

### RDV Inhibited Renal Fibrosis Through Smad7

GS significantly increased the expression of Smad7 at 10 μM in NRK-49F cells or at 1 μM in HK2 cells (Figure 4A). Moreover, RDV significantly enhanced the expression of Smad7 at 1 μM in NRK-49F or at 0.1 μM in HK2 cells (Figure 4B).

The expression of Smad7 was decreased at day 10 after UUO operation in mouse kidneys, which was significantly increased by intraperitoneal injection of RDV (Figure 4C). Similarly, renal injection of RDV significantly increased the expression of Smad7 in UUO mouse kidneys (Figure 4D).

The effect of Smad7 siRNA was tested in NRK-49F cells. Smad7 siRNA significantly reduced the expression of Smad7 and increased the expression of FN and pSmad3 in NRK-49F cells at 24h after transfection (Figure 4E). We further showed that Smad7 siRNA transfection blocked the inhibition of GS on FN and pSmad3 expression in TGF-β treated NRK-49F cells (Figure 4F). Moreover, the inhibitory effect of RDV on FN expression in TGF-β treated NRK-49F cells was blocked by Smad7 siRNA transfection (Figure 4G).

## Discussion

The effect of RDV on renal fibrosis is currently unknown. In the present study, we showed that RDV inhibits renal fibrosis. First, treatment with RDV or its active metabolite GS inhibited fibrosis in two different renal cell lines. Second, systemic administration of RDV inhibited renal fibrosis as shown by Masson staining and Western blotting. Third, local infusion of RDV into UUO kidneys further confirmed the anti-fibrotic effect of RDV.

The limitation of this study is that UUO model is not suitable to study the pharmaceutical effect on renal function. UUO model is a classic model to study renal fibrosis, and we did not observe significant difference in Scr and BUN levels between UUO mice and sham mice (Martinez-Klimova et al., 2019). The renal and liver function assessed in this study were used to exclude the potential toxic effect of RDV, and we show no effect of RDV on renal and liver function in this study. Moreover, future study should also be performed in another model of renal fibrosis such as adriamycin-induced nephropathy to confirm the antifibrotic effect of RDV.

The concentration of RDV was measured to document the efficiency of RDV delivery to the kidney. However, we are not able to detect RDV in serum or kidneys with two different dosing regimens or at different time points. This is probably due to the rapid turnover of RDV by esterases which are abundant in blood and tissues (Warren et al., 2016; Sheahan et al., 2017). Thus, we next measured the concentration of two RDV metabolites, alanine metabolite (Ala-Met) and nucleoside metabolite (GS) (Warren et al., 2016). GS and Ala-Met can be detected at 1 h after i.p. injection in serum and kidneys of sham and UUO mice. For the renal injection, GS was largely detected in kidneys and with a little detection in serum. Moreover, Ala-Met can only be detected in kidneys but not in serum at 1 h after renal injection, suggesting that only a little RDV was leaked from the kidney after the local injection.

The TGF-β/Smad signaling pathway is the major pathogenic mechanism in the development of CKD (Lan, 2011). Genetic knockout of Smad3 alleviated fibrosis in several animal models of CKD, indicating that Smad3 is the key mediator TGF-β/Smad signaling pathway (Zhou et al., 2010). Thus, we hypothesized that RDV attenuated renal fibrosis through Smad3. Indeed, we observed that phosphorylation of Smad3 was attenuated by RDV or its metabolites in two different renal cell lines and in the UUO model.

However, our *in vitro* and *in vivo* data showed that the effect of RDV on Smad3 phosphorylation is weaker than its effect on pro-fibrotic protein production, implying that other fibrotic signaling pathways mediate the anti-fibrotic effect of RDV. Indeed, we found that RDV increased the expression of Smad7, a negative regulator of TGF-β/Smad signaling pathway, *in vitro* and *in vivo*. Moreover, knockdown of Smad7 blocked the anti-fibrotic effect of GS and RDV on renal cells.

In conclusion, we showed that RDV inhibits renal fibrosis in obstructed kidneys, which is correlated with reduced phosphorylation of Smad3 and increased expression of Smad7.

## Data Availability

The raw data supporting the conclusions of this article will be made available by the authors, without undue reservation.
